# Social participation and mental health among university students—a social integration perspective

**DOI:** 10.3389/fpsyg.2025.1654004

**Published:** 2025-10-09

**Authors:** Juan Feng, Jia Li

**Affiliations:** ^1^Mental Health Education Center, Xi’an University of Technology, Xi'an, China; ^2^Mental Health Education Center, Northwest University, Xi'an, China

**Keywords:** university students, social participation, social integration, mental health, influence mechanism

## Abstract

**Objective:**

The transition to university life represents a critical period for mental health, with social participation playing a pivotal role in student adjustment and mental health. This study aims to examine the relationship between social participation and mental health among university students, utilizing Social Integration Theory as a theoretical lens to elucidate how different dimensions of social participation contribute to or detract from psychological well-being within the university social environment. The findings seek to provide nuanced insights into the dynamics of social integration and its implications for student mental health promotion.

**Methods:**

This study employed a quantitative, cross-sectional survey design. All 1,147 participants were undergraduate students from diverse academic disciplines at University H in China. The participants aged between 18 and 23 years, with 783 males (68.3%). The reliability and validity of questionnaire and scales were assessed using Exploratory Factor Analysis and Cronbach’s alpha coefficient. The variables examined included Social Participation, Mental Health, Social Support, and Sense of Belonging, along with demographic variables such as age, gender, economic status and academic year. Statistical analyses, including correlation analysis and mediation analysis, were conducted to examine the associations between social participation and mental health outcomes, controlling relevant covariates.

**Results:**

This study found that university students with a higher economic status tended to participate more frequently in campus group activities and social networking activities, while those with a lower economic status were more likely to participate in social resource-building activities. Freshmen were found to be most active in campus group activities, whereas juniors were more involved in social resource-building activities. Social support and sense of belonging play significant buffering/enhancing roles in the relationship between social participation and depression, life satisfaction.

**Conclusion:**

Social participation plays a crucial role in facilitating university students’ integration into campus life and has a substantial impact on their mental well-being. This study validates the applicability of social integration theory in elucidating the social adaptation process of university students through empirical analysis. On this basis, it further offers a significant theoretical framework and practical guidance for enhancing mental health policies for university students.

## 1 Introduction

University students constitute a significant portion of the resident population in China, with their numbers projected to exceed 48.46 million by 2024 (Ministry of Education of the People’s Republic of China, 2025). This demographic not only symbolizes the nation’s future but also fulfills a crucial role within the broader social framework. Mental health refers to a favorable state of coordination and balance in an individual’s cognitive, emotional, and behavioral dimensions (World Health Organization, 2025). It plays a crucial role in shaping the social adaptability of young people and enhancing their quality of life. It will serve as a critical indicator of the effectiveness of public health and social security systems. However, the mental health of university students is not optimistic. In the World Mental Health Surveys International University Student Project, researchers found that among 13,984 full-time students from 19 universities across 8 countries, roughly 1/3 of the first-year students (who participated in a self-report survey) screened positive for at least one common Diagnostic and Statistical Manual of Mental Disorders–IV (DSM–IV) anxiety, mood, or substance disorder (35.3% lifetime, 31.4% over 12 months) (Auerbach et al., 2018). The Mental Health Blue Book: Report on the Development of National Mental Health in China (2021–2022) released by the Institute of Psychology, Chinese Academy of Sciences indicated that the risk detection rate of depression among university students was approximately 21.48%, while the risk detection rate of anxiety was as high as 45.28% (Fu and Zhang, 2023). Poor mental health of students in higher education have significant implications on academic, social, and economic fronts, leading to academic underachievement and an elevated likelihood of university dropout, which is an increasing concern for public health and policy (Campbell et al., 2022).

Social participation significantly impacts individual physical and psychological health (Barbieri, 2021). University students’ social participation is a key influencing factor for their well-being (Cicognani et al., 2008). Social participation refers to a person’s involvement in activities that provide interaction with others in society or the community (Schröder et al., 2022; Li and Xu, 2024) reflecting the important connection between the individual and their environment. Previous studies suggested that the space for social participation should be external to the family, within the wider societal space (Schröder et al., 2022; Liang and Che, 2023; Lin et al., 2024). Thus, this study centers on the social participation of Chinese university students beyond their dormitory premises, delineating social participation as an individual’s involvement in activities that foster interpersonal interactions. Existing research on social participation predominantly focused on immigrants (Johnson-Singh et al., 2024), elders (Ding et al., 2022), and other groups with social difficulties (such as those with an autism spectrum disorder, etc.) (Dalemans et al., 2010; Orsmond et al., 2013; Tobin et al., 2014). These groups encounter similar integration challenges, and active social participation fosters favorable conditions for their integration and adaptation (Webber and Fendt-Newlin, 2017). Existing literature recognized that the shift from high school to university poses one of the most intricate stages in a student’s academic journey, exposing university students to numerous adaptive hurdles (Sá, 2023). Upon entering university, university students often relocate to a different city, presenting them with the task of acclimating to their new surroundings. The theory of social integration serves as a crucial framework when discussing social adaptation and social integration.

Social integration, a core concept in sociology and psychology, process in which immigrants and their activities become intertwined in social life and form mutually interdependent relations with the receiving society (Elbakidze et al., 2025). It encompasses elements including identity, social acceptance, cultural adaptation, and integration into the socioeconomic status of the host society (Xie et al., 2022; Yang et al., 2025). Social integration has a significant impact on individual physical and mental health (Brydsten et al., 2019; Zhou et al., 2022). Some researchers have divided social integration into four dimensions: political, economic, cultural, and psychological integration (Xie et al., 2022). Psychological integration is considered as the advanced and final stage of social integration (Xie et al., 2022). Psychological integration usually refers to psychological identity, which is the self-perception and emotional belonging to one’s own identity (Xiang, 2023). This study focuses solely on the Psychological integration dimension within social integration. In contrast to the integration of politics, economy, and culture, psychological integration prioritizes emotional bonds at the psychological level and the development of a sense of belonging.

Tinto’s (1993) model of social integration posited that individuals must establish congruence with the subculture of at least one sub-community within an institution to foster a sense of institutional belonging. Inadequate social interactions resulting in a lack of congruence may result in insufficient social support and potentially lead to university withdrawal (Min and Chau, 2012). Thus, positive social interactions and fostering a sense of belonging are pivotal factors for university students to successfully assimilate into the university environment. Therefore, the positive interaction with others and the establishment of a sense of belonging are the key factors for university students to integrate into university. Based on this, the present study attempts to explore how university students’ social participation affects their mental health by promoting social integration.

Social support and sense of belonging are two concepts closely related to social integration (Wilcox et al., 2005; Xie et al., 2022), both belonging to the psychological integration dimension of social integration. Social support refers to the social resources individuals perceive as available or provided by non-professionals within both formal support groups and informal helping relationships (Gottlieb and Bergen, 2010). Existing research suggested that social support was protective against the adverse effects of perceived stress on depression for both Latinx and White university students which were from a designated Hispanic-serving research university in a Southwestern city; however, social support was particularly important for Latinx students in the context of stress to prevent anxiety symptoms (Johnson-Esparza et al., 2021). A meta-analysis of 66 studies by researchers found that social support was closely related to individuals’ anxiety and depression status, with lower social support increasing the risk of depression and anxiety (Wickramaratne et al., 2022). Psychologists described the sense of belonging as a fundamental human motivation (Baumeister and Leary, 1995). For university students in the critical period of socialization, the formation of a sense of belonging was closely related to their mental health (Gopalan and Brady, 2020). Meanwhile, sense of belonging was a key predictor of depression symptoms in adolescents (Parr et al., 2020). In a nationally representative sample of first-year U.S. university students, researchers found that belonging predicts better persistence, engagement, and mental health (Gopalan and Brady, 2020). Thoits (2011) posited that social support (particularly emotional support) directly strengthened an individual’s sense of belonging to a group by fulfilling their companionship needs, thereby facilitating their integration into social networks. A study from China found that university students’ participation in sports activities can effectively enhance their sense of belonging and identity (Ji et al., 2024). Social support and a sense of belonging are fundamental psychological components of social integration, playing a crucial role in determining the level of integration. Thus, the acquisition of social support and a sense of belonging upon entering university is essential for successful integration into campus life for students. Social participation plays a crucial role in fostering social support and a sense of belonging among university students.

China university students present different characteristics from western students under the background of collectivistic culture (Cao and Meng, 2020). First, the campus ecosystem follows a centralized management model: Chinese universities commonly implement a unified on-campus housing and fixed dormitory system, with each dormitory typically housing 4 to 6 students. Under normal circumstances, dormitory compositions remain relatively stable throughout the four-year university period without frequent changes. Moreover, dormitory mates usually belong to the same class, sharing identical course schedules, resulting in largely synchronized daily routines among roommates. Second, academic pressure is relatively high: Chinese universities generally adopt a two-semester system (spring and autumn), with each semester lasting approximately 20 weeks. Chinese university students, especially those in Science, Technology, Engineering, Mathematics (STEM) fields, face substantial academic workloads, averaging 30 class hours per week. Studies showed that Chinese university students spend more than twice as much time on independent academic activities (Shostya, 2015). Third, there is significant individual variation among students. Chinese university students typically come from all 34 province across the country, spanning vast geographical distances and strong cultural diversity, resulting in notable differences in living habits, upbringing backgrounds, and cognitive approaches. The aforementioned distinctions are likely to result in varying social participation traits. However, current research on the social participation characteristics of Chinese university students remains relatively scarce.

This study aims to investigate the impact of social participation on the mental health of university students within the framework of social integration theory. It seeks to gather empirical evidence from the Chinese university student population to inform the development of policies addressing mental health issues among this demographic. The study focuses on three key questions outlined below.

Research question 1: What is the present status and characteristics of social participation among university students?

Research question 2: What is the relationship between university students’ social participation and mental health?

Research question 3: As core factors in social integration, how do social support and sense of belonging influence the relationship between social participation and university students’ mental health?

## 2 Methodology

### 2.1 Participants

This study targeted 3,503 non-psychology undergraduate students from China’s H University of Science and Technology (with a total enrollment of over 28,000), which is located in a central city in China. A total of 1,147 valid questionnaires were finally collected from this survey. The social participation patterns of senior students notably diverge from those of freshmen to juniors, primarily due to internship or graduation thesis obligations. To ensure the findings’ representativeness, this study exclusively examined freshmen to juniors.

The study comprised 1,147 students, with 783 males (68.3%) and 364 females (31.7%), aged between 18 and 23 years, and with an average age of 19.76 years. The distribution among academic years was as follows: 491 freshmen (42.8%), 296 sophomores (25.8%), and 360 juniors (31.4%). The students were enrolled in various engineering disciplines such as Hydraulic Engineering and Electronic Engineering. All participants signed informed consent forms before participating in this research.

### 2.2 Instruments

This research employed a self-designed Social Participation Questionnaire to evaluate the frequency of social participation among university students. Individual mental health was assessed using the Self-Rating Depression Scale, Self-Rating Anxiety Scale, and Satisfaction with Life Scale. Social support was evaluated using the Multidimensional Scale of Perceived Social Support, while the General Belongingness Scale was used to measure the sense of belonging. Demographic variables included gender, age, and academic year. To account for the potential influence of economic status on social participation, the study examined students’ living expenses as a proxy for economic standing. Based on initial interviews, university students’ living expenses ranged from below 1,000 Yuan to over 2,500 Yuan. This study categorizes university students’ economic status into five grades within this range and examines the correlation between various economic levels and levels of social participation.

#### 2.2.1 Social participation questionnaire

This study uses a self-designed questionnaire to evaluate the social participation of Chinese university students. Some researchers classified students’ social participation activities into: involvement in academic activities, involvement in cultural activities, involvement in recreational/sports activities, and involvement in associative activities (Sá, 2023). Based on previous studies and the actual situation of university students’ social participation in China, this study designed a questionnaire with 10 items to measure university students’ social participation. Responses were recorded on a 5-point scale ranging from “never participated” to “once a week or more.” This threshold was selected because a meaningful measure of social participation requires that it occurs with some regularity (Johnson-Singh et al., 2024). In this study, the Cronbach’s *α* coefficient of the questionnaire was 0.763.

The Kaiser-Meyer-Olkin (KMO) test and Bartlett’s test of sphericity were employed to analyze the questionnaire data. The KMO value was 0.825, and Bartlett’s test indicated significance (*p* < 0.001), confirming the suitability of the data for factor analysis. Through factor analysis, the social participation of university students was categorized into three dimensions, reflecting their level of engagement in various activities within the preceding 3 months. Based on the outcomes of the factor analysis and considering the actual context of university students’ social participation, three dimensions were identified: campus group activities, social resource-building activities, and social networking activities. Campus group activities (e.g., “Participate in on-campus cultural and artistic activities such as club events, class activities, singing competitions, sports events like the school sports day, and cultural festivals.”), which center on collaboration and communication, emphasize the frequency of interaction with others during collective activities. Social resource-building activities [e.g., “Part-time social activities (such as tutoring, part-time waitstaff, etc.)”], which focus on accumulating social resources, emphasize the frequency of utilitarian and reciprocal interactions with others. Social networking activities (e.g., “Gathering with school friends and classmates, engaging in alumni exchanges, and other activities.”), which center around emotional communication, emphasize the frequency of emotional interactions with others.

#### 2.2.2 Multidimensional scale of perceived social support

The Multidimensional Scale of Perceived Social Support (SPSS) was developed by Zimet et al. (1988), and was translated and modified by Jiang (2001) to form the Chinese version. The Chinese version of the scale was divided into three dimensions: family support, friend support, and support from other individuals. It assessed an individual’s perceived level of support from family (e.g., “I can discuss my problems with my family”), friends (e.g., “I can rely on my friends when difficulties arise”), and other individuals [e.g., “I can share my joys and sorrows with certain people (supervisors, relatives, colleagues)”]. The total score reflected the individual’s perceived level of social support. This scale was appropriately modified based on the characteristics of university students, with adjustments made to the four items in the dimension of support from others. The terms “leaders, relatives, colleagues” was revised to “teachers, classmates “. An example of the modified item is: “I can share happiness and sadness with some people (teachers, classmates).” This scale employed a 7-point Likert scale, ranging from 1 “completely disagree” to 7 “completely agree,” to quantify individuals’ subjective perceptions of support. Higher scores indicated a stronger perceived support by the individual. In examining the mechanism underlying the association between negative parenting practices among middle school students and school-related fear, the Cronbach’s *α* coefficient for the scale utilized was 0.952 (Guo et al., 2025). In the present study, the Cronbach’s *α* coefficient for the same scale was 0.946. The KMO value was 0.888, and Bartlett’s test indicated significance (*p* < 0.001).

#### 2.2.3 General belongingness scale

The General Belongingness Scale (GBS) was developed by Malone et al. (2012), consisting of 2 dimensions and 12 items. Two of the dimensions were acceptance and inclusion (e.g., “I feel accepted by others”) and rejection and exclusion (e.g., “I feel like an outsider”). This scale adopted a 7-point Likert scale, ranging from 1 “completely disagree” to 7 “completely agree,” to quantify an individual’s sense of belonging level. Deng et al. (2020) translated The General Belongingness Scale into Chinese, obtaining Cronbach’s *α* coefficients ranging from 0.90 to 0.93 for the total scale and its two dimensions, indicating good reliability. In order to adapt to the characteristics of university students, two items in this study were modified accordingly. The original statements “I have close relationships with my family and friends” and “I am not included in the plans of my friends and family” were revised to “I have close relationships with my teachers and classmates” and “I am not included in the plans of friends and classmates at school “In this study, the Cronbach’s *α* coefficient of the scale was 0.95. The KMO value was 0.95, and Bartlett’s test indicated significance (*p* < 0.001).

#### 2.2.4 Self-rating depression scale

The Self-Rating Depression Scale (SDS) was developed by Zung (1965). This study employed the Chinese revised version of the Self-Rating Depression Scale (Zhang, 1993) to assess the participants’ level of depression over the past week. The scale consists of 20 items, using a 4-point scoring method (1 = never or occasionally, 2 = sometimes, 3 = often, 4 = always). The scale includes 10 positively scored items (e.g., “I feel downhearted and low in spirits”) and 10 reverse-scored items (e.g., “I have hope for the future. “). After reverse scoring the negatively worded items, the total raw score is calculated by summing all item scores. Higher scores on the scale correspond to greater levels of depression severity. In a study by Shi et al. (2024) investigating the correlation between parental helicopter parenting and subjective well-being among university students, the scale demonstrated a Cronbach’s *α* coefficient of 0.81. In the present study, the scale exhibited a Cronbach’s *α* coefficient of 0.883. The KMO value was 0.929, and Bartlett’s test indicated significance (*p* < 0.001).

#### 2.2.5 Self-rating anxiety scale

The Self-Rating Anxiety Scale was developed by Zung (1971). This study employed the Chinese revised version of the anxiety self-assessment scale (Zhang, 1993) to evaluate the participants’ anxiety levels over the past week. The scale consists of 20 items, using a 4-point rating method (1 = never or occasionally, 2 = sometimes, 3 = often, 4 = always). Among them, there are 15 positively scored items (e.g., “I feel more nervous and anxious than usual”) and 5 negatively scored items (e.g., “I think everything is fine and no misfortune will happen”). Once reverse scoring has been conducted on the negatively worded items, the total raw score is computed by summing the scores of all items. Higher scores on this scale correspond to higher levels of anxiety. In a study by Yang et al. (2024) on the development of depression and anxiety among Chinese adolescents, the Cronbach’s *α* coefficients of the scale across three longitudinal measurements were 0.86/0.86/0.84. The Cronbach’s α coefficient for this scale in this study was 0.873. The KMO value was 0.936, and Bartlett’s test indicated significance (*p* < 0.001).

#### 2.2.6 Satisfaction with Life Scale

The Satisfaction with Life Scale developed by Diener et al. (1985), was translated and revised into Chinese by Wang et al. (2009). The scale consists of 5 items (e.g., “I am satisfied with my life”), using a 7-point Likert scale ranging from 1 “completely disagree” to 7 “completely agree” to quantify an individual’s life satisfaction. In the study exploring the relationship between gratitude and life satisfaction among university students conducted by Hu et al. (2024), the Cronbach’s α coefficient of the questionnaire was 0.94. This study focused on the characteristics of university students’ life satisfaction and additionally developed five items that align with the actual circumstances of university students (e.g., “Various aspects of my university life correspond with my ideals”). After reliability and validity testing, the Cronbach’s α coefficient for the newly compiled 5 items was 0.93, while the overall Cronbach’s *α* coefficient for the questionnaire was 0.947. The KMO value was 0.948, and Bartlett’s test indicated significance (*p* < 0.001).

### 2.3 Data collection and procedures

In May 2025, 44 questionnaires were administered to freshman students for predictive testing. Following the exclusion of questionnaires with inadequate completion times, excessive completion times, or evident logical inconsistencies, 40 valid questionnaires were retained. Subsequent data analysis involved revising and eliminating ambiguous items, resulting in the establishment of a formal evaluation questionnaire with satisfactory reliability and validity for each scale.

The formal testing was conducted from May to June 2025, employed the Tencent Questionnaire platform and the Wenjuanwang platform to administer electronic surveys. QR codes of this testing were disseminated by researchers to 3,503 university students. These QR codes included pertinent assessments, information on informed consent forms, and links to surveys. To uphold data integrity, each device was limited to a single submission, and completion of all required fields was obligatory. After filtering out responses with excessively brief or prolonged completion times, as well as those displaying clear logical inconsistencies, 1,147 valid questionnaires were retained, resulting in an overall response rate of 32.74%. The study prioritized ensuring participant anonymity and explicitly stated that the data collected would be utilized solely for research purposes, thereby safeguarding personal information.

### 2.4 Data analysis methods

Statistical analysis was performed using SPSS 25.0. A one-way ANOVA was employed to compare the social participation scores of university students with different demographic characteristics. Pearson correlation analysis was employed to examine the correlations between variables. The mediation effects of social support and sense of belonging between social participation and mental health variables were tested using Model 6 in the SPSS Process 4.1 plugin developed by Hayes.

## 3 Results

### 3.1 Demographic variables

The research was carried out at University H in China, with a predominant representation of male participants (68.3%), while freshmen made up 42.8% of the sample. More than half of the university students (51.2%) reported economic status (monthly living expenses) ranging from 1,000 to 1,500 Yuan, indicating a relatively modest standard of living (Considering the local economic context, a monthly budget of 1,000–1,500 Yuan can only cover students’ basic food expenses). As living expenses surpass 1,500 Yuan, university students begin to have progressively more disposable money (see Table 1).

**Table 1:** Descriptive statistics of demographic variables (*N* = 1,147).

Demographic variable	Category	Number of participants	%
Sex	Male	783	68.3%
	Female	364	31.7%
Grade	Freshman	491	42.8%
	Sophomore	296	25.8%
	Junior	360	31.4%
Economic status	Below 1,000 Yuan	75	6.5%
	1,000–1,500 Yuan (inclusive)	587	51.2%
	1,200–2,000 Yuan (inclusive)	371	32.3%
	2,000–2,500 Yuan (inclusive)	85	7.4%
	Above 2,500 Yuan	29	2.5%

### 3.2 Overview of university students’ social participation

University students’ social participation was assessed using a set of 10 items that measure the frequency of engagement in various activities. An exploratory factor analysis, employing principal component analysis, was conducted on these items. Table 2 presented the component matrix after rotation of social participation items. The analysis revealed the presence of three factors, which accounted for a cumulative variance of 59.497% in the original variables.

**Table 2:** Component matrix after rotation of social participation items.

Items	Factor loading		
	Campus group activities	Social resource-building activities	Social networking activities
Item1: Participate in on-campus academic and research activities such as subject competitions, research projects, lectures, and forums.	0.726		
Item2: Participate in on-campus cultural and artistic activities such as club events, class activities, singing competitions, sports events like the school sports day, and cultural festivals.	0.808		
Item3: Participate in on-campus sports activities such as basketball or school events, or other recreational activities like board games.	0.667		
Item5: Participate in on-campus part-time activities (such as teacher assistants).		0.710	
Item6: Out-of-school social practice activities (such as summer social practices, social surveys, etc.)		0.744	
Item7: Out-of-school volunteer activities (community service, teaching assistance programs, volunteer activities, etc.)		0.615	
Item8: Part-time social activities (such as tutoring, part-time waitstaff, etc.)		0.651	
Item9: Out-of-school training and activities (such as vocational skills training, corporate internships, etc.)		0.751	
Item4: Gathering with school friends and classmates, engaging in alumni exchanges, and other activities.			0.709
Item10: Gathering and interacting with non-university friends.			0.794

Table 3 presented Overview of university students’ social participation. In the dimension of social participation, university students had the highest frequency in participating in social networking activities, followed by campus group activities, and the lowest in social resource-building activities. Taking a monthly cycle as the unit, the frequency of social participation for most students was between “less than once a month” and “once a month” (2.3 ± 0.587), suggesting a relatively low frequency of social participation.

**Table 3:** Overview of university students’ social participation (*N* = 1,147).

Items	M ± D	Median	Mode
Item1: Participate in on-campus academic and research activities such as subject competitions, research projects, lectures, and forums.	2.30 ± 0.924	2.00	2
Item2: Participate in on-campus cultural and artistic activities such as club events, class activities, singing competitions, sports events like the school sports day, and cultural festivals.	2.34 ± 0.988	2.00	2
Item3: Participate in on-campus sports activities such as basketball or school events, or other recreational activities like board games.	2.82 ± 1.303	3.00	2
Item4: Gathering with school friends and classmates, engaging in alumni exchanges, and other activities.	3.03 ± 1.138	3.00	3
Item5: Participate in on-campus part-time activities (such as teacher assistants).	1.46 ± 0.831	1.00	1
Item6: Out-of-school social practice activities (such as summer social practices, social surveys, etc.)	1.86 ± 0.799	2.00	2
Item7: Out-of-school volunteer activities (community service, teaching assistance programs, volunteer activities, etc.)	2.03 ± 0.927	2.00	2
Item8: Part-time social activities (such as tutoring, part-time waitstaff, etc.)	1.75 ± 1.046	1.00	1
Item9: Out-of-school training and activities (such as vocational skills training, corporate internships, etc.)	1.58 ± 0.855	1.00	1
Item10: Gathering and interacting with non-university friends.	2.35 ± 1.058	2.00	2
Campus group activities	2.49 ± 0.833	2.33	2
Social resource-building activities	1.73 ± 0.642	1.60	1
Social networking activities	2.69 ± 0.880	2.50	3
Social participation	2.30 ± 0.587	2.28	2

Table 4 presented differences in social participation among university students with varying economic status. The results indicated significant variations in social participation frequencies among university students based on their economic circumstances [*F*(4,1,142) = 5.857, *p* < 0.001]. Notably, disparities in participation rates were evident across campus group activities [*F*(4,1,142) = 2.478, *p* = 0.043], social resource-building activities [*F*(4,1,142) = 3.417, *p* = 0.009], and social networking activities [*F*(4,1,142) = 8.884, *p* < 0.001] among students with different living expense brackets. Students with living expenses between 2,000 and 2,500 Yuan exhibited the highest engagement in campus group activities and social networking activities, while those with expenses below 1,000 Yuan demonstrated the highest involvement in social resource-building activities.

**Table 4:** Differences in social participation among university students with varying economic status (*N* = 1,147).

Variables	Economic status	Means	SD	*F*	*p*
Campus group activities	Below 1,000 Yuan	2.449	0.956	2.478*	0.043
	1,000–1,500 Yuan (inclusive)	2.423	0.789		
	1,200–2,000 Yuan (inclusive)	2.549	0.837		
	2,000–2,500 Yuan (inclusive)	2.671	0.843		
	Above 2,500 Yuan	2.483	1.153		
Social resource-building activities	Below 1,000 Yuan	1.968	0.852	3.417**	0.009
	1,000–1,500 Yuan (inclusive)	1.706	0.581		
	1,200–2,000 Yuan (inclusive)	1.708	0.626		
	2,000–2,500 Yuan (inclusive)	1.802	0.729		
	Above 2,500 Yuan	1.848	0.963		
Social networking activities	Below 1,000 Yuan	2.62	0.989	8.884***	0.000
	1,000–1,500 Yuan (inclusive)	2.56	0.826		
	1,200–2,000 Yuan (inclusive)	2.818	0.858		
	2,000–2,500 Yuan (inclusive)	3.000	0.967		
	Above 2,500 Yuan	2.966	1.172		
Social participation	Below 1,000 Yuan	2.346	0.75	5.857***	0.000
	1,000–1,500 Yuan (inclusive)	2.229	0.551		
	1,200–2,000 Yuan (inclusive)	2.359	0.552		
	2,000–2,500 Yuan (inclusive)	2.491	0.607		
	Above 2,500 Yuan	2.432	0.926		

The degrees of freedom for the *F* value are df_1_ = 4, df_2_ = 1,142. *p < 0.05, **p < 0.01, ***p < 0.001.

Table 5 presented differences in social participation among university students with varying academic year. The findings indicated significant differences in campus group activities and social resource-building activities across various academic years [*F*(2,1,144) = 22.103, *p* < 0.001; *F*(2,1,144) = 15.841, *p* < 0.001]. Specifically, freshmen exhibited significantly higher participation rates in campus group activities compared to sophomores and juniors. Conversely, juniors demonstrated notably greater engagement in social resource-building activities compared to sophomores and freshmen. However, there were no significant variances in the overall social participation scores across academic years [*F*(2,1,144) = 2.05, *p* = 0.129], nor were there significant differences in social networking activities [*F*(2,1,144) = 0.799, *p* = 0.45].

**Table 5:** Differences in social participation among university students with varying academic year (*N* = 1,147).

Variables	Academic year	Means	SD	*F*	*p*
Campus group activities	Freshman	2.664	0.851	22.103***	0.000
	Sophomore	2.413	0.795		
	Junior	2.3	0.79		
Social resource-building activities	Freshman	1.644	0.624	15.841***	0.000
	Sophomore	1.701	0.642		
	Junior	1.887	0.641		
Social networking activities	Freshman	2.704	0.886	0.799	0.45
	Sophomore	2.635	0.849		
	Junior	2.717	0.898		
Social participation	Freshman	2.337	0.591	2.05	0.129
	Sophomore	2.25	0.573		
	Junior	2.301	0.592		

The degrees of freedom for the *F* value are df_1_ = 2, df_2_ = 1,144. *p < 0.05, **p < 0.01, ***p < 0.001.

### 3.3 The correlation between university students’ social participation and mental health

Campus group activities, social resource-building activities, social networking activities and social participation were significantly positively correlated with life satisfaction [*r*(1145) = 0.187, *p* < 0.001; *r*(1145) = 0.094, *p* = 0.01; *r*(1145) = 0.143, *p* < 0.001; *r*(1145) = 0.194, *p* < 0.001]. Campus group activities were significantly negatively correlated with depression [*r*(1145) = −0.157, *p* < 0.001] and anxiety [*r*(1145) = 0.066, *p* = 0.026]. Social networking activities and social participation were significantly negatively correlated with depression [*r*(1145) = −0.100, *p* = 0.01; *r*(1145) = −0.12, *p* < 0.001]. Notably, social resource-building activities were significantly positively correlated with anxiety [*r*(1145) = 0.091, *p* = 0.002] (see Table 6).

**Table 6:** The correlation between university students’ social participation and key variables.

Variables	1	2	3	4	5	6	7	8
1. Campus group activities								
2. Social resource-building activities	0.350***							
3. Social networking activities	0.344***	0.306***						
4. Social participation	0.772***	0.682***	0.774***					
5. Social support	0.205***	0.034	0.163***	0.191***				
6. Sense of belonging	0.226***	0.024	0.196***	0.213***	0.800***			
7. Depression	−0.157***	0.011	−0.100**	−0.120***	−0.619***	−0.725***		
8. Anxiety	−0.066*	0.091**	−0.030	−0.013	−0.491***	−0.597***	0.804***	
9. Life satisfaction	0.187***	0.094**	0.143***	0.194***	0.602***	0.669***	−0.669***	−0.542***

*N* = 1,147, df = 1,145, *p < 0.05, **p < 0.01, ***p < 0.001.

### 3.4 The mediating effect of social support and sense of belonging between social participation and mental health

Model 6 from the SPSS Process 4.1 plugin by Hayes was utilized in this study to investigate the mediating roles of social support and sense of belonging in the relationship between social participation and mental health. Social participation and its various dimensions were considered as the independent variables, while depression, anxiety, and life satisfaction were the dependent variables. Gender, academic year, and Economic status were included as covariates, with social support and sense of belonging serving as the mediating variables.

#### 3.4.1 The chain mediating effect of social support and sense of belonging between campus group activities and depression

Table 7 presented the regression analysis results of the relationship between campus group activities and depression. The results showed that campus group activities had a significant negative predictive effect on depression (*B* = −0.080, *p* < 0.001). When social support and sense of belonging were included in the regression equation, campus group activities significantly predicted social support (*B* = 0.246, *p* < 0.001) and sense of belonging (*B* = 0.069, *p* = 0.002). Social support significantly predicted sense of belonging (*B* = 0.791, *p* < 0.001) and negatively predicted depression (*B* = −0.049, *p* = 0.001). In addition, sense of belonging was a significant negative predictor of depression (*B* = −0.280, *p* < 0.001). At this point, the direct effect value of campus group activities on depression was no longer significant (*B* = 0.006, *p* = 0.594). These results indicated that social support, sense of belonging and the chain mediating effect of social support → sense of belonging were significant among the influences of campus group activities on depression.

**Table 7:** Regression analysis of the relationship between campus group activities and depression (*N* = 1,147).

Regression equation result variable	Predictor variable	Fitting index			Significance	
		*R*	*R* ^2^	*F*	*B*	*t*
Depression		0.176	0.031	9.166***		
	Gender				−0.042	−1.525
	Academic year				−0.002	−0.141
	Economic status				−0.035	2.217*
	Campus group activities				−0.080	−5.051***
Social support		0.255	0.065	19.858***		
	Gender				0.183	2.950**
	Academic year				0.064	1.866
	Economic status				0.127	3.629***
	Campus group activities				0.246	6.970***
Sense of belonging		0.804	0.646	416.142***		
	Gender				0.079	2.049*
	Academic year				−0.038	−1.773
	Economic status				0.007	0.306
	Social support				0.791	43.049***
	Campus group activities				0.069	3.083**
Depression		0.728	0.530	214.657***		
	Gender				0.029	1.507
	Academic year				0.005	0.424
	Economic status				0.001	0.134
	Sense of belonging				−0.280	−18.826***
	Social support				−0.049	−3.271**
	Campus group activities				0.006	0.534

*p < 0.05, **p < 0.01, ***p < 0.001.

**Figure 1:**
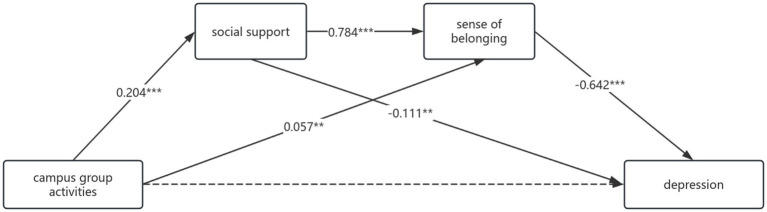
A model illustrating the chain mediating effects of social support and sense of belonging between campus group activities and depression, with all path coefficients being unstandardized *p < 0.05, **p < 0.01, ***p < 0.001.

Table 8 (mediating effect values of social support and sense of belonging between campus group activities and depression) and Figure 1 (chain mediating model of social support and sense of belonging) showed that social support and sense of belonging exerted a significant mediating role between campus group activities and depression, with a total mediating effect value of −0.086. This mediating effect consisted of three indirect paths: 1. Campus group activities → Social support → Depression (−0.012), accounting for 15% of the total effect; 2. Campus group activities → Sense of belonging → Depression (−0.019), accounting for 23.75% of the total effect; 3. Campus group activities → Social support → Sense of belonging → Depression (−0.055), accounting for 68.75% of the total effect. The 95% confidence intervals of all three indirected effects did not include zero, confirming their significance. Bootstrap 95% confidence interval analysis showed no significant difference between indirect effects 1 and 2 (interval includes zero), but significant differences existed between effects 1 and 3, and 2 and 3. These results indicated that campus group activities can indirectly predict depression through three paths: the single mediation of social support, the single mediation of sense of belonging, and the chain mediation of social support → sense of belonging. Among them, the chain mediating effect (68.75%) was significantly higher than the two single mediating effects.

**Table 8:** Mediating effects of social support and sense of belonging between campus group activities and depression (*N* = 1,147).

Path	Effect	SE	95%CI		Relative mediation effect
			LLCI	ULCI	
Campus group activities → Social support → Depression	−0.012	0.004	−0.021	−0.004	15%
Campus group activities → Sense of belonging → Depression	−0.019	0.007	−0.033	−0.006	23.75%
Campus group activities → Social support → Sense of belonging → Depression	−0.055	0.009	−0.073	−0.037	68.75%
Compare 1	0.007	0.008	−0.008	0.023	
Compare 2	0.043	0.009	0.026	0.062	
Compare 3	0.035	0.011	0.014	0.057	

LLCI refers to the lower limit of the 95% confidence interval for the estimated value, and ULCI refers to the upper limit of the 95% confidence interval for the estimated value.

#### 3.4.2 The chain mediating effect of social support and sense of belonging between social networking activities and depression

Table 9 presented the regression analysis results of the relationship between social networking activities and depression. The results showed that social networking activities had a significant negative predictive effect on depression (*B* = −0.045, *p* = 0.003). When social support and sense of belonging were included in the regression equation, social networking activities significantly predicted social support (*B* = 0.166, *p* < 0.001) and sense of belonging (*B* = 0.077, *p* < 0.001). Social support significantly predicted sense of belonging (*B* = 0.793, *p* < 0.001) and negatively predicted depression (*B* = −0.049, *p* = 0.001). In addition, sense of belonging was a significant negative predictor of depression (*B* = −0.283, *p* < 0.001). At this point, social networking activities significantly and positively predicted depression (*B* = 0.023, *p* = 0.031). These results indicated that social support, sense of belonging and the chain mediating effect of social support → sense of belonging were significant among the influences of social networking activities on depression.

**Table 9:** Regression analysis of the relationship between social networking activities and depression (*N* = 1,147).

Regression equation result variable	Predictor variable	Fitting index			Significance	
		*R*	*R* ^2^	*F*	*B*	*t*
Depression		0.131	0.017	4.976**		
	Gender				−0.048	−1.733
	Academic year				0.013	0.845
	Economic status				−0.034	−2.103*
	Social networking activities				−0.045	−2.984*
Social support		0.214	0.046	13.718***		
	Gender				0.201	3.210**
	Academic year				0.018	0.525
	Economic status				0.119	3.319**
	Social networking activities				0.166	4.964***
Sense of belonging		0.804	0.647	418.626***		
	Gender				0.083	2.154*
	Academic year				−0.051	−2.433*
	Economic status				−0.001	−0.047
	Social support				0.793	43.660***
	Social networking activities				0.077	3.734***
Depression		0.730	0.532	216.20***		
	Gender				0.030	1.538
	Academic year				0.003	0.317
	Economic status				−0.001	−0.135
	Sense of belonging				−0.283	−19.014***
	Social support				−0.049	−3.257**
	Social networking activities				0.023	2.157*

*p < 0.05, **p < 0.01, ***p < 0.001.

**Figure 2:**
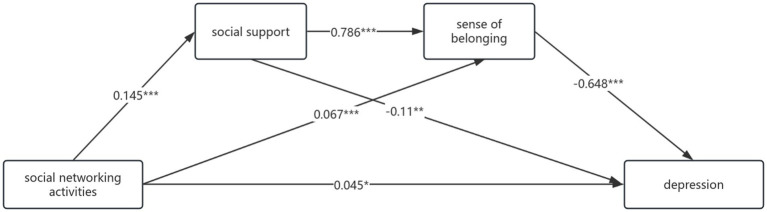
A model illustrating the chain mediating effects of social support and sense of belonging between social networking activities and depression, with all path coefficients being unstandardized *p < 0.05, **p < 0.01, ***p < 0.001.

Table 10 (mediating effect values of social support and sense of belonging between social networking activities and depression) and Figure 2 (chain mediating model of social support and sense of belonging) showed that social support and sense of belonging exerted a significant mediating role between social networking activities and depression, with a total mediating effect value of −0.067. This mediating effect consisted of three indirect paths: 1. Social networking activities → Social support → Depression (−0.008), accounting for 17.78% of the total effect; 2. Social networking activities → Sense of belonging → Depression (−0.022), accounting for 48.89% of the total effect; 3. Social networking activities → Social support → Sense of belonging → Depression (−0.037), accounting for 82.22% of the total effect. The 95% confidence intervals of all three indirect effects did not include zero, confirming their significance. Bootstrap 95% confidence interval analysis showed no significant difference between indirect effects 2 and 3 (interval includes zero), but significant differences existed between effects 1 and 2, and 1 and 3. These results indicated that social networking activities can indirectly predict depression through three paths: the single mediation of social support, the single mediation of sense of belonging, and the chain mediation of social support → sense of belonging. Among them, the chain mediating effect (82.22%) was significantly higher than the two single mediating effects.

**Table 10:** Mediating effects of social support and sense of belonging between social networking activities and depression (*N* = 1,147).

Path	Effect	SE	95%CI		Relative mediation effect
			LLCI	ULCI	
Social networking activities → Social support → Depression	−0.008	0.003	−0.015	−0.003	17.78%
Social networking activities → Sense of belonging → Depression	−0.022	0.006	−0.034	−0.010	48.89%
Social networking activities → Social support → Sense of belonging → Depression	−0.037	0.008	−0.054	−0.022	82.22%
Compare 1	0.014	0.007	0.000	0.029	
Compare 2	0.029	0.008	0.016	0.045	
Compare 3	0.015	0.010	−0.005	0.035	

LLCI refers to the lower limit of the 95% confidence interval for the estimated value, and ULCI refers to the upper limit of the 95% confidence interval for the estimated value.

#### 3.4.3 The chain mediating effect of social support and sense of belonging between campus group activities and life satisfaction

Table 11 presented the regression analysis results of the relationship between campus group activities and life satisfaction. The results showed that campus group activities had a significant positive predictive effect on life satisfaction (*B* = 0.237, *p* < 0.001). When social support and sense of belonging were included in the regression equation, campus group activities significantly predicted social support (*B* = 0.246, *p* < 0.001) and sense of belonging (*B* = 0.069, *p* = 0.002). Social support significantly predicts sense of belonging (*B* = 0.791, *p* < 0.001) and life satisfaction (*B* = 0.199, *p* < 0.001). In addition, sense of belonging was a significant positive predictor of life satisfaction (*B* = 0.549, *p* < 0.001). At this point, campus group activities no longer significantly predicted life satisfaction (*B* = 0.043, *p* = 0.145). These results indicated that social support, sense of belonging and the chain mediating effect of social support → sense of belonging were significant among the influences of campus group activities on life satisfaction.

**Table 11:** Regression analysis of the relationship between campus group activities and life satisfaction (*N* = 1,147).

Regression equation result variable	Predictor variable	Fitting index			Significance	
		*R*	*R* ^2^	*F*	*B*	*t*
Life satisfaction		0.206	0.042	12.632***		
	Gender				0.161	2.385*
	Academic year				0.017	0.447
	Economic status				0.058	1.521
	Campus group activities				0.237	6.173***
Social support		0.255	0.065	19.858***		
	Gender				0.183	2.950*
	Academic year				0.064	1.866
	Economic status				0.127	3.629***
	Campus group activities				0.246	6.970***
Sense of belonging		0.804	0.646	416.142***		
	Gender				0.079	2.049*
	Academic year				−0.038	−1.773
	Economic status				0.007	0.306
	Social support				0.791	43.049***
	Campus group activities				0.069	3.083*
Life satisfaction		0.680	0.462	163.140***		
	Gender				0.001	0.027
	Academic year				−0.003	−0.112
	Economic status				−0.026	−0.916
	Sense of belonging				0.549	14.120***
	Social support				0.199	5.103***
	Campus group activities				0.043	1.458

*p < 0.05, **p < 0.01, ***p < 0.001.

**Figure 3:**
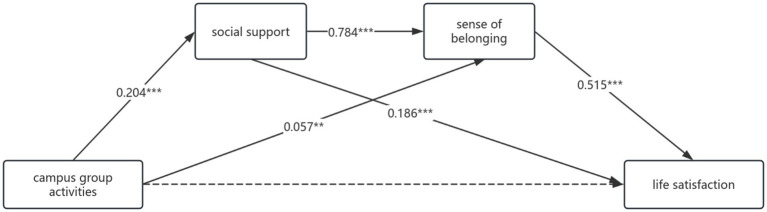
A model illustrating the chain mediating effects of social support and sense of belonging between campus group activities and life satisfaction, with all path coefficients being unstandardized *p < 0.05, **p < 0.01, ***p < 0.001.

Table 12 (mediating effect values of social support and sense of belonging between campus group activities and life satisfaction) and Figure 3 (chain mediating model of social support and sense of belonging) showed that social support and sense of belonging exerted a significant mediating role between campus group activities and life satisfaction, with a total mediating effect value of 0.194. This mediating effect consisted of three indirect paths: 1. Campus group activities → Social support → Life satisfaction (0.049), accounting for 20.68% of the total effect; 2. Campus group activities → Sense of belonging → Life satisfaction (0.038), accounting for 16.03% of the total effect; 3. Campus group activities → Social support → Sense of belonging → Life satisfaction (0.107), accounting for 45.15% of the total effect. The 95% confidence intervals of all three indirect effects did not include zero, confirming their significance. Bootstrap 95% confidence interval analysis showed no significant difference between indirect effects 1 and 2 (interval includes zero), but significant differences existed between effects 1 and 3, 2 and 3. These results indicated that campus group activities can indirectly predict life satisfaction through three paths: the single mediation of social support, the single mediation of sense of belonging, and the chain mediation of social support → sense of belonging. Among them, the chain mediating effect (45.15%) was significantly higher than the two single mediating effects.

**Table 12:** Mediating effects of social support and sense of belonging between campus group activities and life satisfaction (*N* = 1,147).

Path	Effect	SE	95%CI		Relative mediation effect
			LLCI	ULCI	
Campus group activities → Social support → Life satisfaction	0.049	0.014	0.025	0.079	20.68%
Campus group activities → Sense of belonging → Life satisfaction	0.038	0.014	0.011	0.065	16.03%
Campus group activities → Social support → Sense of belonging → Life satisfaction	0.107	0.019	0.072	0.147	45.15%
Compare 1	0.011	0.021	−0.030	0.052	
Compare 2	−0.058	0.021	−0.104	−0.019	
Compare 3	−0.069	0.023	−0.115	−0.027	

LLCI refers to the lower limit of the 95% confidence interval for the estimated value, and ULCI refers to the upper limit of the 95% confidence interval for the estimated value.

#### 3.4.4 The chain mediating effect of social support and sense of belonging between social networking activities and life satisfaction

Table 13 presented the regression analysis results of the relationship between social networking activities and life satisfaction. The results showed that social networking activities had a significant positive predictive effect on life satisfaction (*B* = 0.167, *p* < 0.001). When social support and sense of belonging were included in the regression equation, social networking activities significantly predicts social support (*B* = 0.166, *p* < 0.001) and sense of belonging (*B* = 0.077, *p* < 0.001). Social support significantly predicts sense of belonging (*B* = 0.793, *p* < 0.001) and life satisfaction (*B* = 0.202, *p* < 0.001). In addition, sense of belonging was a significant positive predictor of life satisfaction (*B* = 0.551, *p* < 0.001). At this point, social networking activities no longer significantly predict life satisfaction (*B* = 0.018, *p* = 0.517). These results indicated that social support, sense of belonging and the chain mediating effect of social support → sense of belonging were significant among the influences of social networking activities on life satisfaction.

**Table 13:** Regression analysis of the relationship between social networking activities and life satisfaction (*N* = 1,147).

Regression equation result variable	Predictor variable	Fitting index			Significance	
		*R*	*R* ^2^	*F*	*B*	*t*
Life satisfaction		0.169	0.028	8.347***		
	Gender				0.178	2.623*
	Academic year				−0.028	−0.753
	Economic status				0.049	1.254
	Social networking activities				0.167	4.598***
Social support		0.214	0.046	13.718***		
	Gender				0.201	3.210*
	Academic year				0.018	0.525
	Economic status				0.119	3.319*
	Social networking activities				0.166	4.964***
Sense of belonging		0.804	0.647	418.626***		
	Gender				0.083	2.154*
	Academic year				−0.051	−2.433*
	Economic status				−0.001	−0.047
	Social support				0.793	43.660***
	Social networking activities				0.077	3.734***
Life satisfaction		0.679	0.461	162.612***		
	Gender				0.004	0.071
	Academic year				−0.011	−0.406
	Economic status				−0.027	−0.921
	Sense of belonging				0.551	14.144***
	Social support				0.202	5.182***
	Social networking activities				0.018	0.648

*p < 0.05, **p < 0.01, ***p < 0.001.

**Figure 4:**
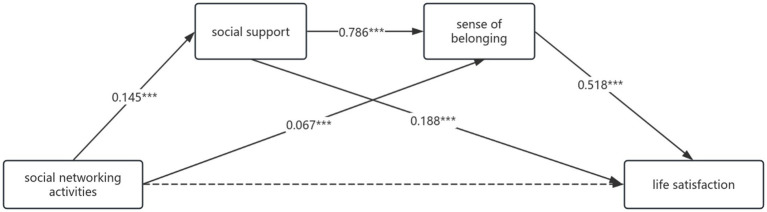
A model illustrating the chain mediating effects of social support and sense of belonging between social networking activities and life satisfaction, with all path coefficients being unstandardized *p < 0.05, **p < 0.01, ***p < 0.001.

Table 14 (mediating effect values of social support and sense of belonging between social networking activities and life satisfaction) and Figure 4 (chain mediating model of social support and sense of belonging) showed that social support and sense of belonging exerted a significant mediating role between social networking activities and life satisfaction, with a total mediating effect value of 0.149. This mediating effect consisted of three indirect paths: 1. Social networking activities → Social support → Life satisfaction (0.034), accounting for 20.36% of the total effect; 2. Social networking activities → Sense of belonging → Life satisfaction (0.043), accounting for 25.75% of the total effect; 3. Social networking activities → Social support → Sense of belonging → Life satisfaction (0.073), accounting for 43.71% of the total effect. The 95% confidence intervals of all three indirect effects did not include zero, confirming their significance. Bootstrap 95% confidence interval analysis showed no significant difference between indirect effects 1 and 2, 2 and 3 (interval includes zero), but significant differences existed between effects 1 and 3. These results indicated that social networking activities can indirectly predict life satisfaction through three paths: the single mediation of social support, the single mediation of sense of belonging, and the chain mediation of social support → sense of belonging. Among them, the chain mediating effect (43.71%) was significantly higher than the two single mediating effects.

**Table 14:** Mediating effects of social support and sense of belonging between social networking activities and life satisfaction (*N* = 1,147).

Path	Effect	SE	95%CI		Relative mediation effect
			LLCI	ULCI	
Social networking activities → Social support → Life satisfaction	0.034	0.011	0.015	0.058	20.36%
Social networking activities → Sense of belonging → Life satisfaction	0.043	0.013	0.018	0.069	25.75%
Social networking activities → Social support → Sense of belonging → Life satisfaction	0.073	0.016	0.042	0.106	43.71%
Compare 1	−0.009	0.019	−0.045	0.029	
Compare 2	−0.039	0.015	−0.072	−0.012	
Compare 3	−0.030	0.020	−0.069	0.008	

LLCI refers to the lower limit of the 95% confidence interval for the estimated value, and ULCI refers to the upper limit of the 95% confidence interval for the estimated value.

## 4 Discussion

This study assessed the extent of social participation among university students in China through a self-constructed questionnaire, elucidating the features and current state of their social participation. Additionally, adopting a social integration framework, the study investigated the correlation between social participation and mental health, uncovering the intermediary function of social support and sense of belonging in linking social participation to mental health outcomes.

### 4.1 Characteristics of social participation among Chinese university students

This study established a reliable and valid questionnaire to assess social participation among university students. Existing research on social participation predominantly centered on elderly (Xiao et al., 2021; Min et al., 2016) and immigrant populations (Johnson-Singh et al., 2024). The measurement items for social participation primarily derived from extensive surveys like the China Health and Retirement Longitudinal Study (CHARLS) national survey (Xiao et al., 2021) and the China General Social Survey (CGSS) (Liang, 2024). Alternatively, some studies employed the retrospective 24-h diary method (Wang and Cheng, 2024) or self-developed items (Johnson-Singh et al., 2024) to gauge social participation. However, there was no standardized tool available for measuring social participation comprehensively. This study aims to create an assessment tool specifically designed for evaluating the social participation of university students. The tool developed in this study will serve as a valuable resource for future research endeavors focusing on the social participation of university students.

Based on findings from the self-developed questionnaire, university students participated most frequently in social networking activities, followed by campus group activities, with the lowest engagement in social resource-building activities. Existing studies indicated that individuals in China demonstrated significantly stronger collectivist awareness than those in Western countries (Oyserman et al., 2002). Within this cultural context, university students tended to value collective participation—both social interactions and campus group activities essentially involved individual-collective interaction, through which personal values were often explored and affirmed. Moreover, fostering strong friendships was crucial for adapting to university life, as these relationships typically served as students’ primary source of social support (Wilcox et al., 2005); strong identification with university friendship groups most effectively mitigated distress compared to other social identities, with this protective effect mediated by reduced loneliness (McIntyre et al., 2018). Notably, the significantly low participation rate in social resource-building activities stemmed from multiple factors: physical distance between activity venues and students’ residences affected participation frequency (Sá, 2023), and substantial entry barriers—such as quantitative limitations and access requirements for many resource-accumulation opportunities (e.g., prioritizing financially disadvantaged students for certain part-time positions)—inhibited broader involvement.

The economic status significantly influenced the social participation of university students. Students with better financial means tended to participate more frequently in campus group activities and social networking activities, as indicated by previous research (Munir et al., 2023). Higher economic status affords students increased social interaction opportunities and the necessary financial support for their participation in social activities. Conversely, students facing financial constraints are more inclined to actively seek resources both within and outside the campus, such as through part-time work and internships, to secure financial support.

The academic year significantly influenced the social participation of university students. The first year of university was a critical stage for students to negotiate their identity as new members of the university community and to build a sense of belonging, which contributed to better adjustment to university life (Min and Chau, 2012). Previous studies found that active participation in extracurricular activities had an important impact on students’ career development (Han and Kwon, 2018). This study further confirmed that juniors pivoted towards accruing social capital and bolstering career competitiveness to lay the groundwork for future advancement. This shift in priorities prompted corresponding modifications in their social participation, highlighting notable variations in social participation based on academic year.

### 4.2 The relationship between social participation and mental health

This study revealed a significant association between social participation and the life satisfaction and mental well-being of university students. It was important to highlight that distinct forms of social participation exhibit varying degrees of correlation with the mental health of university students.

Campus group activities and social networking activities were positively correlated with life satisfaction, and negatively correlated with anxiety and depression. Prior research indicated that meaningful social participation can provide valuable social and emotional support for students during their adaptation to university life (Maunder, 2018). Campus group activities and social networking activities were more interactive. In the process of in-depth interaction with others, they facilitated the establishment of mutual aid resources, enhanced individuals’ positive emotional experiences, and promoted individual mental health. It was noteworthy that social resource-building activities was positively correlated with the anxiety of university students. Social resource-building activities (such as internships, part-time jobs, and social practices) were essentially a competition for limited resources (such as internship positions, part-time job opportunities, and skill certificates). Posselt and Lipson (2016) found that high levels of perceived competition associated with increased risks of anxiety. Competition-induced stress can lead to anxiety among university students, while simultaneously hindering their ability to seek support from participants, thereby exacerbating individual anxiety levels.

### 4.3 Social participation influences university students’ mental health through social support and sense of belonging

Based on the pathways through which social participation influenced mental health, social support and sense of belonging played significant buffering/enhancing roles in the relationship between social participation and mental health. On the one hand, university students’ social participation enhanced their sense of belonging (Min and Chau, 2012) and identity through interactions with others (Ji et al., 2024), gaining attention, acceptance, and assistance from others (social support) (Rhubart and Kowalkowski, 2022). An individual’s perceived social support and sense of belonging in interactions can effectively buffer the negative psychological impact of stressful events and reduce depression (Johnson-Esparza et al., 2021; Wickramaratne et al., 2022). Increased social support further facilitated the formation of a sense of belonging: when individuals perceived support from others, they were more likely to develop the psychological identification of belonging to this group. This was potentially because emotional support from friends provided a sense of belonging and can also help students face problems (Wilcox et al., 2005). The formation of a sense of belonging further promoted individual mental health (Begen and Turner-Cobb, 2015). On the other hand, social integration emphasized individuals establishing close and frequent social relationships and interactions within society, encompassing emotional bonds and the transmission of shared values. More people interacting, more frequent interaction, and talking about personal topics may increase social support (Han, 2023). Low-frequency social participation made it difficult to establish emotional connections and obtain psychological support.

Notably, this study identified an important phenomenon through regression analysis: after controlling for social support and sense of belonging, the predictive effect of social networking activities on depression shifted from significantly negative to positive, and this shift is consistent with the characteristics of a typical suppression effect. This indicates that not all uses of social networking activities yield psychological benefits—only high-quality interactions that truly enhance emotional connection and sense of belonging exert a positive effect on mental health, whereas low-quality social networking interactions may expose its potential mental health risks. This finding is consistent with the conclusions of Kuczynski et al. (2022). Thus, the essence of the impact of university students’ social participation on mental health lies in: strengthening emotional connections with others through positive interactions, perceiving social support and a sense of belonging, and ultimately enhancing mental health levels.

### 4.4 Theoretical reflections on the research findings

Social integration theory was instrumental in elucidating the correlation between university students’ social participation and mental health. Previous studies on social integration theory focused on immigrant groups (Brydsten et al., 2019; Zhou et al., 2022; Yang et al., 2025). Social integration theory emphasized that psychological integration was the final stage of individual social integration, representing the true integration of individuals into new societies (Xie et al., 2022) and affecting individual mental health (Brydsten et al., 2019; Zhou et al., 2022). Drawing parallels between university students and immigrants in encountering similar societal transitions, this study delved into elucidating the intrinsic workings of university students’ social participation on their mental health through a social integration perspective.

Contrary to previous studies (Yen et al., 2022; Liang, 2024), this study found that social participation did not directly improve individual mental health. But consistent with Brydsten et al. (2019), this study underscored the significance of social support and a sense of belonging as pivotal determinants for individuals to effectively assimilate into unfamiliar settings. This study confirmed the core idea of social integration theory. Furthermore, this research built upon Brydsten’s work by delineating the internal mechanisms through which social participation influenced an individual’s mental health. It delves into the internal processes through which university students acclimate to the university environment via social involvement upon commencing their academic journey, thereby fostering their mental health.

This study not only extended the scope of social integration theory but also provided empirical evidence for its application in young student populations, offering both theoretical innovation and practical educational implications.

### 4.5 Limitation and further research

This study has certain limitations. First, the cross-sectional nature of the data hindered the study from making inferences beyond a static analytical framework, failing to reveal the dynamic causal relationships between variables such as university students’ social participation and mental health over time. Second, the exploration of potential influencing factors was insufficient, with many dimensions of influence and action pathways remaining unknown—these underlying mechanisms hidden beneath the surface associations needed to be excavated.

Given the aforementioned constraints, future research should focus on advancing two key areas. First, methodologically, adopting a longitudinal tracking design with periodic data collection and developing time series models would enable a comprehensive analysis of the dynamic relationships and causal mechanisms among variables. Second, in exploring influencing factors, an integrated approach combining quantitative and qualitative methods is critical. Qualitative techniques such as interviews and focus groups can yield in-depth insights, which, when triangulated with statistical analyses of quantitative data, would facilitate a rigorous examination of the underlying mechanisms of potential influencing factors—thereby advancing the field holistically.

## 5 Conclusion

Social participation plays a crucial role in facilitating university students’ integration into campus life and has a substantial impact on their mental well-being. This study delineated the concept and various dimensions of social participation among university students, culminating in the creation of a reliable and valid questionnaire to assess their social participation. The inception of this instrument not only furnishes a standardized means for subsequent quantitative inquiries but also establishes a scientific underpinning for evaluating university students’ mental health and shaping relevant policies.

This study validates the applicability of social integration theory in elucidating the social adaptation process of university students through empirical analysis. The research identifies that key components of social integration theory, such as social support and sense of belonging, play a crucial mediating role in the social participation and mental well-being of university students. It is revealed that social participation alone does not directly enhance mental health; rather, its impact is contingent upon factors like psychological integration, perceived social support, and a sense of belonging. These findings systematically elucidate how university students establish positive interactions within the campus environment and social circles through social participation.

This study offers a significant theoretical framework and practical guidance for enhancing mental health policies for university students. Through data analysis, the following recommendations are proposed. For university administrators: incorporate students’ social participation into campus life integration assessments, increase resource allocation to social participation platforms, establish records for tracking activity involvement and mental health. For local educational authorities: facilitate regional universities’ exchange of social participation resources, establish inter-school collaboration platforms, promote sharing of best practices in students’ social participation mechanisms, and support campus social network expansion. For health and education policy agencies: drawing on the social integration theoretical framework, emphasize the significance of enhancing students’ social participation in mental health policy development for university students. Simultaneously, introduce supportive policies to encourage partnerships between universities and communities, expand campus social activities to community services, and intensify efforts in providing social support for students’ mental well-being.

## Data Availability

The raw data supporting the conclusions of this article will be made available by the authors, without undue reservation.
