# Supplementary material for: Social participation and mental health among university students—a social integration perspective
Source: Front Psychol. 2025 Oct 9;16:1654004. doi: 10.3389/fpsyg.2025.1654004 (PMC12546318; doi:10.3389/fpsyg.2025.1654004)
Supplement: Supplementary file 1 [file Data_Sheet_1.pdf]

University Students' Social Participation and Mental Health Questionnaire

Dear Participants:

We sincerely appreciate your participation in this survey, which is designed to examine the correlation between social participation and mental health among university students. All responses will be utilized exclusively for academic research purposes and shall be treated with the utmost confidentiality. It is important to note that there are no standardized correct or incorrect answers; thus, we kindly request that you provide responses based on your genuine experiences and perceptions. The completion of this questionnaire is estimated to take approximately 10 minutes. Your valuable support and cooperation are highly appreciated.

Part I: Demographic surveys

1. Your gender:

① Male

② Female

2. Your age (fill in the blanks):

3. The school you belong to:

① School of Mechanical and Precision Instrument Engineering

② School of Automation and Information Engineering

③ School of Science

④ School of Civil Engineering and Architecture

⑤ School of Water Resources and Hydropower Engineering

⑥ School of Materials Science and Engineering

⑦ School of Electrical Engineering

4. Your grade:

① freshman year

② sophomore

③ Junior year

5. Your monthly living expenses:

①Below 1000 yuan

②1000-1500 yuan (inclusive)

③1500-2000 yuan (inclusive)

④2000-2500 yuan (inclusive)

⑤More than 2500 yuan

Part II: Questionnaire items (no scale name is presented during formal testing)

This questionnaire consists of six parts. Please read each statement carefully, answer according to your actual feelings, and choose the one that best suits your actual situation.

1.Social participation questionnaire

This questionnaire consists of 10 items. Please respond based on how frequently you have participated in various activities over the past three months.

(1) On-campus academic and research activities (e.g., subject competitions, research projects, lectures, forums, etc.)

① Never participated

② Less than once a month

③ Once a month

④ Approximately twice a month

⑤ Once a week or more

(2) On-campus cultural and artistic activities (e.g., club activities, class activities, art events like singing competitions, sports activities like sports meets, cultural events like campus cultural festivals, etc.)

① Never participated

② Less than once a month

③ Once a month

④ Approximately twice a month

⑤ Once a week or more

(3) On-campus sports activities (e.g., basketball, sports meets, etc.) or other games and recreational activities (e.g., board games, etc.)

① Never participated

② Less than once a month

③ Once a month

④ Approximately twice a month

⑤ Once a week or more

(4) Activities such as gatherings with on-campus friends and classmates, alumni exchanges, etc.

① Never participated

② Less than once a month

③ Once a month

④ Approximately twice a month

⑤ Once a week or more

(5) On-campus part-time activities (e.g., student assistant roles, etc.)

① Never participated

② Less than once a month

③ Once a month

④ Approximately twice a month

⑤ Once a week or more

(6) Off-campus social practice activities (e.g., summer social practice, social surveys, etc.)

① Never participated

② Less than once a month

③ Once a month

④ Approximately twice a month

⑤ Once a week or more

(7) Off-campus volunteer service activities (e.g., community service, teaching assistance, volunteer programs, etc.)

① Never participated

② Less than once a month

③ Once a month

④ Approximately twice a month

⑤ Once a week or more

(8) Social part-time activities (e.g., tutoring, part-time waiter/waitress roles, etc.)

① Never participated

② Less than once a month

③ Once a month

④ Approximately twice a month

⑤ Once a week or more

(9) Off-campus training and learning activities (e.g., vocational skills training, corporate internships, etc.)

① Never participated

② Less than once a month

③ Once a month

④ Approximately twice a month

⑤ Once a week or more

(10) Activities such as gatherings and interactions with non-university friends, etc.

① Never participated

② Less than once a month

③ Once a month

④ Approximately twice a month

⑤ Once a week or more

2.Multidimensional scale of perceived social support

This scale consists of 12 items. Please answer them according to your actual situation.

(11) When I encounter problems, some people (teachers, classmates) will be by my side.

① Completely disagree

② Quite disagree

③ Slightly disagree

④ Neutral

⑤ Slightly agree

⑥ Quite agree

⑦ Completely agree

(12) I can share happiness and sadness with some people (teachers, classmates).

① Completely disagree

② Quite disagree

③ Slightly disagree

④ Neutral

⑤ Slightly agree

⑥ Quite agree

⑦ Completely agree

(13) My family can provide me with practical and specific help.

① Completely disagree

② Quite disagree

③ Slightly disagree

④ Neutral

⑤ Slightly agree

⑥ Quite agree

⑦ Completely agree

(14) I can get emotional help and support from my family when needed.

① Completely disagree

② Quite disagree

③ Slightly disagree

④ Neutral

⑤ Slightly agree

⑥ Quite agree

⑦ Completely agree

(15) When I am in trouble, some people (teachers, classmates) are a true source of comfort for me.

① Completely disagree

② Quite disagree

③ Slightly disagree

④ Neutral

⑤ Slightly agree

⑥ Quite agree

⑦ Completely agree

(16) My friends can really help me.

① Completely disagree

② Quite disagree

③ Slightly disagree

④ Neutral

⑤ Slightly agree

⑥ Quite agree

⑦ Completely agree

(17) I can rely on my friends when I am in trouble.

① Completely disagree

② Quite disagree

③ Slightly disagree

④ Neutral

⑤ Slightly agree

⑥ Quite agree

⑦ Completely agree

(18) I can talk about my problems with my family.

① Completely disagree

② Quite disagree

③ Slightly disagree

④ Neutral

⑤ Slightly agree

⑥ Quite agree

⑦ Completely agree

(19) My friends can share happiness and sadness with me.

① Completely disagree

② Quite disagree

③ Slightly disagree

④ Neutral

⑤ Slightly agree

⑥ Quite agree

⑦ Completely agree

(20) There are certain people (teachers, classmates) in my life who care about my feelings.

① Completely disagree

② Quite disagree

③ Slightly disagree

④ Neutral

⑤ Slightly agree

⑥ Quite agree

⑦ Completely agree

(21) My family is willing to help me make various decisions.

① Completely disagree

② Quite disagree

③ Slightly disagree

④ Neutral

⑤ Slightly agree

⑥ Quite agree

⑦ Completely agree

(22) I can discuss my problems with my friends.

① Completely disagree

② Quite disagree

③ Slightly disagree

④ Neutral

⑤ Slightly agree

⑥ Quite agree

⑦ Completely agree

3.General belongingness scale

This scale consists of 12 items. Please answer them according to your actual situation.

(23). When I am with others, I feel that I am part of the group

① Completely disagree

② Quite disagree

③ Slightly disagree

④ Neutral

⑤ Slightly agree

⑥ Quite agree

⑦ Completely agree

(24). I have close relationships with teachers and classmates

① Completely disagree

② Quite disagree

③ Slightly disagree

④ Neutral

⑤ Slightly agree

⑥ Quite agree

⑦ Completely agree

(25). I feel accepted by others

① Completely disagree

② Quite disagree

③ Slightly disagree

④ Neutral

⑤ Slightly agree

⑥ Quite agree

⑦ Completely agree

(26). I have a sense of belonging

① Completely disagree

② Quite disagree

③ Slightly disagree

④ Neutral

⑤ Slightly agree

⑥ Quite agree

⑦ Completely agree

(27). When I am with friends, I feel that I have a place among them

① Completely disagree

② Quite disagree

③ Slightly disagree

④ Neutral

⑤ Slightly agree

⑥ Quite agree

⑦ Completely agree

(28). I feel connected to others

① Completely disagree

② Quite disagree

③ Slightly disagree

④ Neutral

⑤ Slightly agree

⑥ Quite agree

⑦ Completely agree

(29). I feel like an outsider

① Completely disagree

② Quite disagree

③ Slightly disagree

④ Neutral

⑤ Slightly agree

⑥ Quite agree

⑦ Completely agree

(30). I feel that others don’t care about me

① Completely disagree

② Quite disagree

③ Slightly disagree

④ Neutral

⑤ Slightly agree

⑥ Quite agree

⑦ Completely agree

(31). Despite having a sense of belonging, I feel lonely during holidays

① Completely disagree

② Quite disagree

③ Slightly disagree

④ Neutral

⑤ Slightly agree

⑥ Quite agree

⑦ Completely agree

(32). I feel isolated from the world

① Completely disagree

② Quite disagree

③ Slightly disagree

④ Neutral

⑤ Slightly agree

⑥ Quite agree

⑦ Completely agree

(33). When I am with others, I feel like a stranger

① Completely disagree

② Quite disagree

③ Slightly disagree

④ Neutral

⑤ Slightly agree

⑥ Quite agree

⑦ Completely agree

(34). I am not included in the plans of friends and classmates at school

① Completely disagree

② Quite disagree

③ Slightly disagree

④ Neutral

⑤ Slightly agree

⑥ Quite agree

⑦ Completely agree

4. Self-Rating depression scale

This scale consists of 20 items. Please respond based on your actual situation over the past week.

(35). I feel downhearted and low in spirits.

① Never or occasionally

② Sometimes

③ Often

④ Always

(36). I think the morning is the best part of the day.

① Never or occasionally

② Sometimes

③ Often

④ Always

(37). I have crying spells or feel like crying.

① Never or occasionally

② Sometimes

③ Often

④ Always

(38). I have trouble sleeping at night.

① Never or occasionally

② Sometimes

③ Often

④ Always

(39). I eat as much as usual.

① Never or occasionally

② Sometimes

③ Often

④ Always

(40). I feel as happy as before when having close contact with the opposite sex.

① Never or occasionally

② Sometimes

③ Often

④ Always

(41). I notice that my weight is dropping.

① Never or occasionally

② Sometimes

③ Often

④ Always

(42). I am troubled by constipation.

① Never or occasionally

② Sometimes

③ Often

④ Always

(43). My heart beats faster than usual.

① Never or occasionally

② Sometimes

③ Often

④ Always

(44). I feel tired for no reason.

① Never or occasionally

② Sometimes

③ Often

④ Always

(45). My mind is as clear as usual.

① Never or occasionally

② Sometimes

③ Often

④ Always

(46). I don’t find it difficult to do the things I usually do.

① Never or occasionally

② Sometimes

③ Often

④ Always

(47). I feel restless and cannot calm down.

① Never or occasionally

② Sometimes

③ Often

④ Always

(48). I have hope for the future.

① Never or occasionally

② Sometimes

③ Often

④ Always

(49). I get angry or irritated more easily than usual.

① Never or occasionally

② Sometimes

③ Often

④ Always

(50). I find it easy to make decisions.

① Never or occasionally

② Sometimes

③ Often

④ Always

(51). I feel that I am a useful person and that someone needs me.

① Never or occasionally

② Sometimes

③ Often

④ Always

(52). My life is meaningful.

① Never or occasionally

② Sometimes

③ Often

④ Always

(53). I think others would live better if I were dead.

① Never or occasionally

② Sometimes

③ Often

④ Always

(54). I am still interested in the things that usually interest me.

① Never or occasionally

② Sometimes

③ Often

④ Always

5. Self-rating anxiety scale

This scale consists of 20 items. Please respond based on your actual situation over the past week.

(55). I feel more nervous or anxious than usual

① Never or occasionally

② Sometimes

③ Often

④ Always

(56). I feel scared for no reason

① Never or occasionally

② Sometimes

③ Often

④ Always

(57). I tend to feel agitated or panicked

① Never or occasionally

② Sometimes

③ Often

④ Always

(58). I feel like I might go crazy

① Never or occasionally

② Sometimes

③ Often

④ Always

(59). I think everything is fine and no misfortune will happen

① Never or occasionally

② Sometimes

③ Often

④ Always

(60). My hands and feet shake

① Never or occasionally

② Sometimes

③ Often

④ Always

(61). I am troubled by headaches, neck pain, and back pain

① Never or occasionally

② Sometimes

③ Often

④ Always

(62). I feel easily weak and fatigued

① Never or occasionally

② Sometimes

③ Often

④ Always

(63). I feel calm and can easily sit quietly

① Never or occasionally

② Sometimes

③ Often

④ Always

(64). I feel my heart beating fast

① Never or occasionally

② Sometimes

③ Often

④ Always

(65). I am troubled by bouts of dizziness

① Never or occasionally

② Sometimes

③ Often

④ Always

(66). I have fainting spells or feel like I am going to faint

① Never or occasionally

② Sometimes

③ Often

④ Always

(67). I find it easy to breathe in and out

① Never or occasionally

② Sometimes

③ Often

④ Always

(68). My hands and feet feel numb and tingly

① Never or occasionally

② Sometimes

③ Often

④ Always

(69). I am troubled by stomachaches and indigestion

① Never or occasionally

② Sometimes

③ Often

④ Always

(70). I often need to urinate

① Never or occasionally

② Sometimes

③ Often

④ Always

(71). My hands and feet are usually dry and warm

① Never or occasionally

② Sometimes

③ Often

④ Always

(72). I feel my face flushing and getting hot

① Never or occasionally

② Sometimes

③ Often

④ Always

(73). I fall asleep easily and sleep well all night

① Never or occasionally

② Sometimes

③ Often

④ Always

(74). I have nightmares

① Never or occasionally

② Sometimes

③ Often

④ Always

6. Satisfaction with life scale

This scale consists of 10 items. Please answer them according to your actual situation.

(75). For the most part, my college life is close to an ideal state.

① Completely disagree

② Quite disagree

③ Slightly disagree

④ Neutral

⑤ Slightly agree

⑥ Quite agree

⑦ Completely agree

(76). My college life is in a good state

① Completely disagree

② Quite disagree

③ Slightly disagree

④ Neutral

⑤ Slightly agree

⑥ Quite agree

⑦ Completely agree

(77). I am satisfied with my college life

① Completely disagree

② Quite disagree

③ Slightly disagree

④ Neutral

⑤ Slightly agree

⑥ Quite agree

⑦ Completely agree

(78). So far, I have obtained what I consider to be the most important things in college life

① Completely disagree

② Quite disagree

③ Slightly disagree

④ Neutral

⑤ Slightly agree

⑥ Quite agree

⑦ Completely agree

(79). If I could live my life over again, I wouldn’t want to change anything

① Completely disagree

② Quite disagree

③ Slightly disagree

④ Neutral

⑤ Slightly agree

⑥ Quite agree

⑦ Completely agree

(80). My college life matches my ideals in many aspects

① Completely disagree

② Quite disagree

③ Slightly disagree

④ Neutral

⑤ Slightly agree

⑥ Quite agree

⑦ Completely agree

(81). Up to now, my college life has made me feel the meaning and fulfillment of life

① Completely disagree

② Quite disagree

③ Slightly disagree

④ Neutral

⑤ Slightly agree

⑥ Quite agree

⑦ Completely agree

(82). I like my college life

① Completely disagree

② Quite disagree

③ Slightly disagree

④ Neutral

⑤ Slightly agree

⑥ Quite agree

⑦ Completely agree

(83). The experiences gained from college life have filled me with confidence to adapt to the challenges of the future society

① Completely disagree

② Quite disagree

③ Slightly disagree

④ Neutral

⑤ Slightly agree

⑥ Quite agree

⑦ Completely agree

(84). College life makes me feel warm and fulfilled

① Completely disagree

② Quite disagree

③ Slightly disagree

④ Neutral

⑤ Slightly agree

⑥ Quite agree

⑦ Completely agree

Thank you for your participation
